# LRRK2 at the Crossroad of Aging and Parkinson’s Disease

**DOI:** 10.3390/genes12040505

**Published:** 2021-03-29

**Authors:** Eun-Mi Hur, Byoung Dae Lee

**Affiliations:** 1 Department of Neuroscience, College of Veterinary Medicine, Research Institute for Veterinary Science and BK21 Four Future Veterinary Medicine Leading Education & Research Center, Seoul National University, Seoul 08826, Korea; ehur1@snu.ac.kr; 2 Department of Physiology, Kyung Hee University School of Medicine, Seoul 02447, Korea; 3 Department of Neuroscience, Kyung Hee University, Seoul 02447, Korea

**Keywords:** Parkinson’s disease, aging, LRRK2, mitochondria, ROS, autophagy, lysosome

## Abstract

Parkinson’s disease (PD) is a heterogeneous neurodegenerative disease characterized by the progressive loss of dopaminergic neurons in the substantia nigra pars compacta and the widespread occurrence of proteinaceous inclusions known as Lewy bodies and Lewy neurites. The etiology of PD is still far from clear, but aging has been considered as the highest risk factor influencing the clinical presentations and the progression of PD. Accumulating evidence suggests that aging and PD induce common changes in multiple cellular functions, including redox imbalance, mitochondria dysfunction, and impaired proteostasis. Age-dependent deteriorations in cellular dysfunction may predispose individuals to PD, and cellular damages caused by genetic and/or environmental risk factors of PD may be exaggerated by aging. Mutations in the *LRRK2* gene cause late-onset, autosomal dominant PD and comprise the most common genetic causes of both familial and sporadic PD. LRRK2-linked PD patients show clinical and pathological features indistinguishable from idiopathic PD patients. Here, we review cellular dysfunctions shared by aging and PD-associated *LRRK2* mutations and discuss how the interplay between the two might play a role in PD pathologies.

## 1. Introduction

Parkinson’s disease (PD) is one of the most common neurodegenerative diseases found in the elderly population and has been characterized by cardinal clinical manifestations, such as tremor, rigidity, postural instability, and bradykinesia. Microscopically, PD is featured by the presence of proteinaceous inclusions known as Lewy bodies (LBs) and Lewy neurites, which are highly immunoreactive for the protein α-synuclein [1]. One of the key neuropathological features of PD is the progressive degeneration of nigrostriatal dopaminergic (DA) innervation, which is responsible for the core motor symptoms. However, neurodegeneration is not restricted to nigral DA neurons but occurs in many other brain regions as well. PD affects the central, peripheral, and autonomic nervous system and causes heterogenous clinical symptoms, including various and often early presenting nonmotor deficits, which makes PD a heterogeneous, multisystem disorder [2]. To date, a number of mechanisms underlying the onset and progression of PD have been proposed, and most, if not all, theories agree that PD is caused by multiple genetic and environmental factors that degenerate DA neurons in the substantia nigra (SN) [3]. Accumulating lines of evidence suggest that oxidative stress, mitochondrial dysfunction, and abnormal protein clearance associated with the dysfunction of the ubiquitin-proteasome system (UPS) and autophagy-lysosomal systems (ALS) play an important part in PD pathogenesis. Of note, all of these defects are also involved in aging [4], raising the question of how rather general defects that can occur in any type of cell cause PD. It has been suggested that PD may be a local expression of aging on a particular population of cells, which have characteristics that make them highly vulnerable to aging factors [5]. Aging affects other cellular populations, whose defects may cause the heterogeneous, non-motor PD symptoms when combined with other PD risk factors.

The median age of onset for PD is around 60 years and the prevalence increases exponentially over the age of 60 [6]. The majority of PD cases are classified as idiopathic; the causes are unknown but believed to develop from accumulated gene-environment interactions. PD is classified on the basis of phenotype (PD only or PD plus syndromes), age at onset (juvenile or late-onset), and mode of inheritance (autosomal dominant, recessive, or X-linked) [6]. Around 10–15% of all PD shows a family history and about 5% tends to show a Mendelian inheritance [7]. Since the first identification of a disease-segregated missense mutation in the *SNCA* gene [8], a total of 23 loci and 19 genes have been associated with PD. Among the genes, mutations in the *LRRK2* gene encoding leucine-rich repeat kinase 2 (LRRK2) protein comprise the most frequent mutations found in both familial and sporadic PD patients. Clinical features of LRRK2-associated PD patients are indistinguishable from those of idiopathic PD patients [9], but some variations have been reported in neuropathology [10,11,12]. Of note, the penetrance of G2019S mutation, the most common pathogenic variant of LRRK2-associated PD, is variable, but progressively increases with age [13,14]. In this review, we discuss the possible interaction between LRRK2 and other factors associated with aging, with a particular emphasis on how their interplay might affect the onset and progression of PD.

## 2. Aging, LRRK2, and PD

### 2.1. Aging and PD

Aging can be defined as the time-dependent, progressive decline of diverse physiological functions in the individual, leading to increased vulnerability to death and diseases [5,15]. Evidence so far suggests that the rate of aging is controlled, at least to some extent, by genetic and environmental factors [15]. The association of PD with aging has been well appreciated for several decades. Advancing age unequivocally confers the major risk for the development of PD. Aging is required to manifest the symptoms of PD even in carriers born with the dominantly inherited, disease-causing mutations. In the United States, it has been estimated that PD is present in 0.02% of the population who died between 45 and 54 years old and in 8.77% of the population who died after the age of 85 [5]. This age-dependent prevalence of PD, showing more than a 400-fold increase as a function of aging, is much more prominent compared to other age-related diseases, such as cardiovascular diseases and Alzheimer’s disease. Several lines of evidence suggest that advancing age also influences the clinical manifestation and disease progression of PD. In several cross-sectional or longitudinal studies, PD patients with old-age onset showed a faster progression of motor signs or disability [16,17,18,19], decreased levodopa responsiveness [20,21,22], more severe gait and postural impairment [23], more severe cognitive impairment, and increased risk of developing dementia [24,25,26]. 

**Figure 1:**
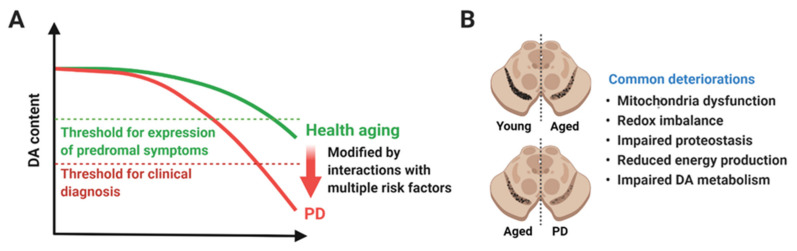
A hypothetical model of aging and Parkinson’s disease (PD). (**A**) Functional DA neurons are reduced in both healthy aging and PD. Rate of degeneration in healthy aging and PD might be similar up to a certain time point or age, but degeneration in PD is accelerated until reaching the threshold for clinical diagnosis (red dotted line). The upper green dotted line represents the threshold for expression of prodromal symptoms. Several genetic and environmental risk factors might play a role in accelerating degeneration of DA neurons and causing PD. (**B**) Degeneration of DA neurons in the SN is considered as the pathological hallmark of PD, but it also occurs in healthy aging. The difference in healthy aging and PD might be the quantity of neurodegeneration rather than the quality. Healthy aging and PD share a plethora of cellular dysfunctions, such as mitochondrial dysfunction, redox imbalance, impaired proteostasis, reduced energy production, and impaired DA metabolism. Created with BioRender.com.

DA neurons in the SN may be particularly vulnerable to aging compared to neurons in other brain regions, such as the hippocampus, putamen, hypothalamus, and neocortex [4,27,28]. The number of cortical neurons in the neocortex was reduced by 9.5% over the range of 70 years (range 20–90 years) [29], whereas the number of DA neurons in the SN of older individuals was reduced by 36.2% compared to younger subjects [30], and the decline was estimated to occur at a rate of 4.7–9.8% per decade [31,32]. A large-scale analysis of post-mortem brains from 744 deceased participants (mean age of death, 88.5 years) without PD revealed that almost 40% of the cases showed mild or more severe DA neuronal loss or the presence of LBs, or both in the SN. As expected, the presence of LBs and the degree of DA neuronal loss were associated with the severity of global parkinsonism and individual parkinsonian signs [33]. Considering that clinical signs of PD are presented when about more than 50% of DA neurons in the SN and 70% of their synaptic terminals in the striatum are lost [5,34,35,36,37], it is plausible that the loss of DA neurons that occur during normal aging is accelerated by a synergistic interaction between age-related changes and other genetic and/or environmental risk factors of PD. Early studies suggested that the quality (i.e., cell-type or location) of degenerating neurons might be different in normal aging versus PD [38], but more recent evidence support the notion that the main difference between aged brains and PD might be the quantity or the extent of DA neuronal loss (Figure 1). 

### 2.2. Characteristics of SN Neurons

**Figure 2:**
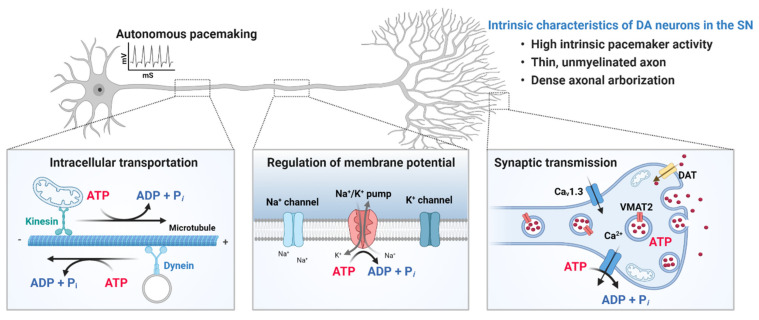
Intrinsic properties of dopaminergic (DA) neurons in the substantia nigra (SN). The nigrostriatal DA neurons are autonomous pacemakers, and their tonic spontaneous activity is important for the sustained release of dopamine in target structures, such as the striatum. In addition, DA neurons in the SN have thin, unmyelinated axons with an extraordinary large axonal arbor and a very high number of synaptic terminals. The size and complexity of DA neurons in the SN impose a high metabolic burden to produce ATP, which is required for transporting cellular components to appropriate locations, maintaining resting membrane potential, generating and propagating action potential, and regulating synaptic transmission. Metabolically active DA neurons in the SN are susceptible to mitochondrial dysfunction, which is a prominent feature of both aging and PD. Created with BioRender.com.

Degeneration of SN neurons in normal aging [39] might be attributed to the vulnerability of their intrinsic properties (Figure 2). The nigrostriatal DA neurons have thin, unmyelinated axons [40] with a dense axonal arborization and a very high number of synaptic terminals [41]. The size and complexity of DA neurons in the SN are orders of magnitude greater than those of other neuronal populations in the brain [42], imposing a high metabolic burden to produce ATP, which is required for maintaining resting membrane potential, generating and propagating action potential, transporting cellular components to appropriate local compartments, and regulating synaptic transmission. Because DA neurons in the SN are metabolically active and the level of basal oxidative phosphorylation is high [43], they are susceptible to mitochondrial dysfunction. Inhibition of mitochondria complex I of the electron transport chain by 1-methyl-4-phenylpyridinium (MPP+) and rotenone induces neurodegeneration of DA neurons in the SN and causes rapid parkinsonism [44,45]. Moreover, activity of complex I and the expression level of complex I subunits were found to be selectively down-regulated in the brains of idiopathic PD patients [46,47,48,49,50]. Defects in mitochondria quality and function, such as reduced activity of mitochondrial enzymes, decreased respiratory capacity per mitochondria, and increased generation of reactive oxygen species (ROS) within SN DA neurons have been implicated in normal aging [51,52] and are similar to those found in postmortem PD brains. The difference again between aged and PD brains might be a matter of the degree of damage, rather than the type. 

Given that mitochondria are a primary source of ROS, production of ROS is greater in neuronal populations with higher energy demands. Due to the gradual deterioration in cellular redox regulatory mechanisms, ROS accumulates in the aging SN. Antioxidants, such as superoxide dismutase (SOD) and glutathione peroxidase play a critical role to reduce ROS and protect cells from pathogenic oxidation. Reduction in SOD and glutathione reductase activities have been reported in the postmortem SN of healthy aged individuals compared to younger individuals [52]. Compared to a rather moderate age-dependent decline in antioxidant activities, severe reduction in glutathione activity has been reported in the SN of PD patients [52,53,54,55]. Thus, a high baseline level of oxidative stress in the aging SN can confer vulnerability to oxidative insults caused by additional compounding factors that are specifically present in the PD brain. 

DA itself can be a source of oxidative stress. DA outside the synaptic vesicle is easily metabolized by monoamine oxidase, generating H_2_O_2_ and dihydroxyphenylacetic acid. Non-enzymatical auto-oxidation of DA produces O_2_^−^ and reactive DA quinone [5,56,57,58]. In DA neurons, iron levels are high, and H_2_O_2_ reacting with iron can form •OH. The DA transporter (DAT) and vesicular monoamine transporter 2 (VMAT2) play a protective role against ROS by removing free dopamine from the synaptic cleft and packing into synaptic vesicles, respectively [59]. Positron emission tomography (PET) imaging with a radiolabeled DAT ligand, N-3-fluoropropyl-2-β-carboxymethoxy-3-β-(4-iodophenyl) nortropane (FPCIT) revealed age-dependent decline in dopamine transporter binding in normal subjects and a significant reduction in idiopathic PD patients [60,61,62]. The decline could reflect a reduction in the number of presynaptic terminals due to cell death and/or a decrease in dopamine transporter availability. Age-related decline in dopamine transporter binding was greater than the age-dependent loss of nigrostriatal neurons in normal aged brain [31], suggesting that the expression of dopamine transporter also decreases with advancing age. Indeed, a marked, age-dependent reduction in dopamine transporter mRNA was reported in human SN [63].

### 2.3. LRRK2 and PD

*LRRK2* encodes a large, multi-domain protein of 2527 amino acids, consisting of a catalytic core and several protein–protein interaction domains. The catalytic core is comprised of a serine/threonine kinase domain and a GTPase domain termed Roc (Ras of complex proteins) followed by the C-terminal of ROC (COR) domain, which classify LRRK2 as a ROCO family protein. The enzymatic core is flanked by additional domains with predicted protein-protein interaction functions, such as the armadillo and ankyrin repeats and the leucin-rich repeat domain at the N-terminus and the WD40 domain at the C-terminus [64]. The N- and the C-terminal regions are suggested to play a regulatory role in controlling enzymatic activity or substrate specificity [65,66]. PD-linked pathogenic LRRK2 mutations are enriched in the catalytic core; R1441C/G/H, N1437H in the ROC domain, Y1699C in the COR domain, and G2019S and I2020T in the kinase domain [67]. Many of the pathogenic LRRK2 mutants display an increase in the kinase activity compared to wild-type (WT) LRRK2 [68,69,70], and genetic or pharmacological inhibition of the kinase activity could alleviate neurodegeneration induced by pathogenic LRRK2 mutants [71], suggesting that aberrant kinase activity plays a key role in the pathogenesis of PD [64,72]. 

Mutations in the *LRRK2* gene can cause autosomal dominant PD [73]. In certain ethnic groups, *LRRK2* mutations have been estimated to be present in up to 40% of familial PD and 10% of sporadic PD cases [74,75,76,77]. The most common LRRK2 mutation, p.G2019S has been reported in many ethnic backgrounds and is estimated to account for 4% of familial PD and 1% of sporadic PD cases [13,74,78]. The frequency of this mutation varies in different ethnic groups [79,80,81,82,83,84]. Core features of LRRK2 G2019S-associated PD include asymmetrical, tremor-predominant parkinsonism with bradykinesia, and rigidity that respond to dopamine replacement and functional neurosurgery [13]. Given that these symptoms are reminiscent of idiopathic PD, LRRK2 G2019S-associated PD and idiopathic PD might share key pathological mechanisms [85]. 

All monogenic variants show heterogeneity in pathology, including the age at onset, disease penetrance, and neuropathological features, such as occurrences of typical LB pathology and/or pure nigral-striatal degeneration. This heterogeneity suggests that the interaction of a certain genetic risk factor with other genetic and/or environmental modifiers affects the neurodegenerative trajectories of PD pathobiology [86]. In the case of LRRK2 G2019S, the penetrance varies widely and increases with age [13,14]. A case-control study conducted by the International LRRK2 Consortium, which included 1,045 people with LRRK2 mutations in 133 families, estimated that the cumulative risk of PD for LRRK2 G2019S carriers was 28% at 59 years, 51% at 69 years, and 74% at 79 years of age [13]. These clinical observations are recapitulated in genetic animal models, presenting highly age-dependent pathological phenotypes. In conditional transgenic (Tg) mice overexpressing LRRK2 G2019S in catecholaminergic neurons, DA neuronal loss could be detected at 15 months of age and progressed until 24 months of age, when deficits in motor function, such as decreased stride length in gait analysis and increased descending time in pole test, became evident [87]. Furthermore, α-synuclein pathology progresses with age. LRRK2 G2019S knock-in (KI) mice exhibited progressive dysfunctions of plasma membrane and vesicular DA transporters between 3 and 12 months of age, along with the increase in phospho-Ser129 α-synuclein-positive inclusions in the striatum compared to age-matched WT mice [88]. However, these pathologies were not associated with nigro-striatal DA neuronal degeneration or changes in striatal DA release at least up to 19 months of age. LRRK2 R1441G mice also showed age-dependent, progressive accumulation of oligomeric α-synuclein in the striatum and the cortex [89]. Differences between LRRK2 R1441G KI mice and age-matched control mice could be detected at 15 months of age and became greater at 18 months of age. Collectively, evidence from LRRK2-associated PD case studies and LRRK2 animal models support the notion that aging is a critical factor for PD development. However, it should be noted that not all studies have not found the same pathologic changes in LRRK2 mouse models. To test the role of aging in PD, Cooper et al. [90] investigated whether delaying aging could suppress LRRK2 G2019S- and α-synuclein A53T-mediated PD phenotypes in *Caenorhabditis elegans* (*C. elegans*) models. They crossed the Tg worm models of PD expressing either LRRK2 G2019S or α-synuclein A53T with the long-lived insulin-IGF1 receptor mutant, *daf-2* and found that the *daf-2* mutation increased the lifespan in both PD mutants. Crossing with *daf-2* mutants also rescued the degeneration of DA neurons and improved DA-dependent behavioral deficit, such as basal slowing, ethanol avoidance response, and area-restricted searching, in the two worm models of PD.

## 3. LRRK2 in Mitochondrial Dysfunctions

### 3.1. LRRK2 and Oxidative Stress

A growing body of evidence supports a role of LRRK2 in mitochondria function. Several aspects of mitochondrial dysfunction, such as increased oxidative stress, reduced mitochondria membrane potential, abnormal mitochondrial fission and fusion, and defects in mitochondrial trafficking, have been suggested from postmortem analysis of LRRK2-linked PD patient tissues [91] and from induced pluripotent stem cells (iPSCs)-derived neural cells from PD patients with LRRK2 mutation [92]. Animal and cellular models expressing pathogenic mutants of LRRK2 also show mitochondrial dysfunction [93]. ROS are generated as byproducts of the respiratory chain reaction in mitochondria and thus oxidative stress is increased in aged and PD brains. An imbalance between ROS production and the ability to detoxify the reactive intermediates can cause oxidative stress, which can create a hazardous state that leads to cell and tissue damage through oxidation of various biological products, such as proteins, lipids, and DNA [94]. 

LRRK2 has been implicated in the regulation of oxidative stress. Oxidative stress in mouse SN-derived SN4741 cells expressing LRRK2 G2019S, LRRK2 WT, or empty vector increased intracellular ROS levels and caused cell death in the order of G2019S > WT > vector-transfected cell [95]. In another report, LRRK2 G2019S and I2020T mutations increased ROS production, and LRRK2 G2019S induced oxidative modification of macromolecules [96]. ROS level and cell death were increased in neural stem cells (NSCs) carrying the R1441G mutation compared to WT NSCs, but was reduced in LRRK2 KO NSCs [97]. Differential gene expression profiling from WT and LRRK2-KO NSCs revealed that several genes involved in oxidation and reduction in mitochondria were deregulated when LRRK2 was depleted [97]. Pathogenic LRRK2 mutations have also been associated with mitochondrial dysfunction. Deficits in mitochondria respiration and compromised mitochondria dynamics were observed in iPSC-derived neural cells from individuals carrying the LRRK2 G2019S or R1441C mutation, but not in iPSC-derived neural cells from healthy subjects [92]. Notably, mitochondrial dysfunction could be rescued by LRRK2 kinase inhibitor. Mitochondrial DNA damage was detected in iPSC-derived neural cells from patients carrying the LRRK2 G2019S mutation [98,99], and zinc finger nuclease-mediated editing of the genetic mutation prevented the mitochondrial DNA damage [99]. The underlying molecular mechanisms by which LRRK2 regulates oxidative stress remain unclear, but the involvement of antioxidant defense mechanisms have been suggested. LRRK2 G2019S could phosphorylate peroxiredoxin 3 (PRDX3) and PRDX2, which is a member of the thioredoxin peroxidase family, and which is an important antioxidant scavenger of hydrogen peroxide in mitochondria. PRDX3 and PRDX2 phosphorylation mediated by LRRK2 was associated with decreased peroxidase activity and increased cell death [96,100,101]. Co-expression of PRDX3 with LRRK2 G2019S in *Drosophila* could ameliorate the reduction in peroxidase activity, loss of DA neurons, shortened lifespan, and mitochondrial defects in flight muscles induced in monogenic flies expressing LRRK2 G2019S alone [100]. Furthermore, LRRK2 G2019S mutation has been associated with mitochondrial uncoupling [102], characterized by dissociation between mitochondrial membrane potential generation and its use for mitochondria-dependent ATP synthesis [103]. LRRK2 G2019S increased mitochondrial proton leak through the upregulation of uncoupling protein (UCP) 2 and UCP4 [94]. These results imply that LRRK2 G2019S increases the permeability of the mitochondrial inner membrane to protons, which can be driven by opening of the permeability pore, increasing the expression of pore forming proteins, and/or the upregulation/activation of UCPs. Opening of mitochondrial permeability pore is associated with increased ROS production [104]. 

Pathogenic LRRK2 mutants have been shown to be associated with increased susceptibility to oxidative stress in multiple model systems [92,95,99,105,106,107,108,109,110]. In several reports, iPSC-derived neural cells from LRRK2 G2019S carriers showed increased vulnerability to oxidative stress caused by exposure to hydrogen peroxide, 6-hydroxydopamine, valinomycin, concanamycin, and MPP+ as compared to iPSC-derived neural cells from healthy individuals [92,107,108], and the increased vulnerability could be reversed by LRRK2 kinase inhibitors [92,108]. In *C. elegans* models, the LRRK2 G2019S mutation increased sensitivity to oxidative and heat stress by inhibiting nuclear translocation of DAF-16, a homolog of mammalian FoxO [111]. FoxO has been suggested as a key transcription factor that coordinates cellular responses to environmental changes, including metabolic and oxidative stress [112]. FoxO can trigger various cellular responses to control the redox status of a cell by regulating the expression of anti-oxidative stress genes, such as sod-3 and dod-3. In *C. elegans*, LRRK-2 G2019S could also exacerbate degeneration of DA neurons caused by exposure to a bacterial metabolite [113]. Similarly, *Drosophila* models expressing dLRRK (*Drosophila* orthologue of hLRRK2) Y1383C (hLRRK2 Y1699C), dLRRK I1915T (hLRRK2 I2020T), hLRRK2 G2019S, or hLRRK2 G2385R showed a significantly higher sensitivity to oxidative stress induced by H_2_O_2_, paraquat, or rotenone, and a marked reduction of DA neurons [109,110]. In a mouse model, DA neurons in the SN pars compacta (SNpc) of LRRK2 G2019S Tg showed increased susceptibility to 1-methyl-4-phenyl-1,2,3,6-tetrahydrophyridine (MPTP) [114,115]. Furthermore, a sub-toxic dose of MPTP caused a severe motor impairment, selective loss of DA neurons in the SNpc, and increased astrocyte activation in LRRK2 G2019S Tg mice, whereas LRRK2 WT Tg mice had mild deficits and non-Tg mice were largely unaffected [115]. A previous study examined the combined effects of LRRK2 mutation, aging, and chronic exposure to an environmental toxin, rotenone [116], and found that LRRK2 R1441G KI mice developed greater locomotor deficits in an open field test compared to WT mice, after oral administration of low doses of rotenone given twice weekly over 50 weeks (half of their lifespan). The increased locomotor deficit was associated with a reduction in striatal mitochondrial complex-I subunit. 

### 3.2. LRRK2 and Mitochondrial Dynamics

Mitochondria are dynamic organelles that constantly fuse and divide, move along cytoskeletal tracks, and undergo regulated turnover. Mitochondria dynamics enables mitochondria quality control and is considered as an important mechanism to adapt to changes in bioenergetic demands and other physiological requirements. During the past decades, core components of a machinery mediating mitochondrial fusion and fission have been identified [117]. Dynamin-related GTPase, mitofusin 1 (MFN1) and its paralog MFN2 are anchored to the outer mitochondrial membrane (OMM) through their C-terminal membrane binding domains and fuse adjacent mitochondrial membrane through N-terminal cytoplasmic regions containing the GTPase domain [118,119,120]. Fusion of the OMMs is driven by GTP hydrolysis, which induces a conformational change to bring the adjacent membranes in contact with one another. Inner mitochondria membrane (IMM) fusion is known to be controlled by another dynamin–related GTPase, optic atrophy protein 1 (OPA1) [121]. OPA1 is processed to generate two forms: the long, membrane-bound OPA1 (L-OPA1) and the proteolytically cleaved short, soluble OPA1 (S-OPA1). The cleavage is mediated by two IMM proteases, YME1L and OMA1 [122]. OPA1 processing is known to be affected by changes in mitochondrial membrane potential and pro-apoptotic stimuli [123,124,125]. Recent studies have suggested that maintaining a delicate balance between L-OPA1 and S-OPA1 isoforms is critical for mitochondrial membrane fusion and remodeling [126]. Similar to OMM fusion by MFN proteins, OPA1 forms oligomeric structures with those of adjacent IMM, followed by GTP hydrolysis-driven conformational change to fuse the two IMMs [127]. Mitochondrial fission is the functional counterpart of fusion, but relatively little is known about molecular mechanisms. Dynamin-related protein 1 (DRP1) is a key molecule in fission, and other proteins such as fission protein 1 (Fis1) and RAB7 have been suggested to play a role [128]. DRP1 localizes in the cytosol and is recruited to the prospective fission site upon activation. 

Structural defects in mitochondria have been reported in various pathogenic LRRK2 models, including LRRK2 G2019S mouse models [129,130,131], LRRK2 PD patient-derived cells [91,132,133,134], and other cellular systems [135,136,137]. Mitochondrial elongation and abnormal interconnectivity have been observed in the fibroblasts derived from LRRK2 G2019S PD patients [91], and abnormally shaped mitochondria have been detected in the striatum of aged LRRK2 G2019S KI mice. Mitochondria observed in the LRRK2 G2019S KI mice resemble “beads-on-a-string”, which show swollen areas with perturbed cristae connected with narrow membranous segments, reminiscent of DRP1-related fission arrest [131]. 

**Figure 3:**
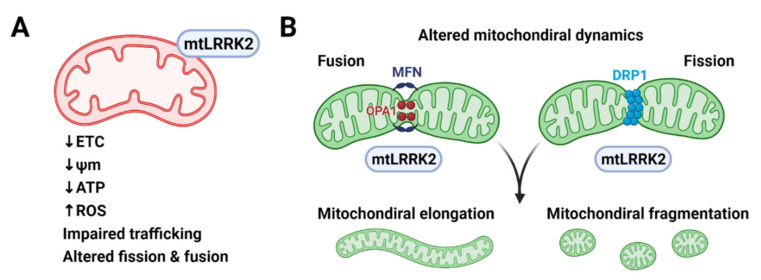
Mitochondrial dysfunction caused by pathogenic LRRK2 mutants. (**A**) Pathogenic LRRK2 mutants (mtLRRK2) reduce the activity of electron transfer chain (ETC) reaction and mitochondria membrane potential (ψm), resulting in inefficient ATP production and increased reactive oxygen species (ROS) production. (**B**) Pathogenic LRRK2 mutants interact with mitochondria fusion proteins, such as mitofusin (MFN) and optic atrophy protein 1 (OPA1) and mitochondria fission proteins, such as dynamin-related protein 1 (DRP1). Pathogenic LRRK2 mutants alter mitochondrial morphology and dynamics presumably by interacting with the fusion and fission machinery but the exact molecular mechanism awaits to be elucidated. Created with BioRender.com (accessed on 13 February 2021).

LRRK2 has been shown to interact with multiple components of the mitochondrial fusion and fission machinery (Figure 3). LRRK2 partially co-localized with MFN1, MFN2 and OPA1 at mitochondrial membranes of neural cells, and WT and PD-associated mutants of LRRK2 (R1441C, Y1699C, and G2019S) could be co-immunoprecipitated with MFN1 [138]. Moreover, LRRK2 has been shown to recruit DRP1 to mitochondria and induce mitochondrial fragmentation in a DRP1-dependent manner. PD-associated mutations (R1441C and G2019S) increased both the mitochondrial recruitment of DRP1 and fragmentation in SH-SY5Y cells and primary cortical neurons [136]. Notably, LRRK2-induced cytotoxicity could be blocked by decreasing mitochondrial fission or increasing fusion [136]. Similar results were observed in PD patient-derived fibroblasts, where LRRK2 G2019S mutation promoted mitochondrial fission by recruiting DRP1 to mitochondria [137]. Interaction between LRRK2 and DRP1 has been also reported in microglial cells [139]. LRRK2 promoted mitochondrial fission by increasing DRP1 expression and triggered a pro-inflammatory response in microglia in LRRK2 G2019S Tg mouse brains [139]. These results suggest that LRRK2 regulates mitochondrial fusion and fission and that dysregulation of mitochondrial dynamics might play a role in LRRK2-associated PD.

## 4. Aging and LRRK2 in Abnormal Protein Clearance

### 4.1. Aging and Protein Aggregation

Proteostasis refers to the state of a balanced proteome and is maintained through the action of the proteostasis network that coordinates protein synthesis, folding, disaggregation, and degradation [140,141]. Age-dependent deterioration of the proteostasis network is regarded as a major driver of age-related cellular dysfunction [15]. Loss of proteostasis is often characterized by the appearance of non-native protein aggregates, which are prominent hallmarks of aging and several neurodegenerative diseases [15,142].

Molecular chaperones function to assist de novo protein folding, prevention of protein misfolding and aggregation, and targeting unfolded and non-native proteins for degradation. Coupled with chaperone functions, two principle proteolytic systems, UPS and ALS play key roles in protein quality control, ensuring the removal of damaged or misfolded proteins. In vitro and in vivo studies have demonstrated that components of the proteostasis network are negatively affected during aging. Poor cellular energetics due to reduced mitochondrial function and dysregulation of cellular metabolism can limit the amount of available ATP, thereby affecting ATP-dependent chaperones and ATP-dependent proteolytic machinery. Age-dependent decline of UPS function may also result from decreased expression of chaperone and proteasome subunits, disassembly of proteasome, and inactivation of accumulated protein aggregates [143,144]. Age-related modifications in the substrate can also interfere with each machinery’s ability to recognize its target [140]. Regarding ALS, autophagic decline contributes to the accumulation of dysfunctional cytoplasmic organelles, such as lysosomes, mitochondria, and endoplasmic reticulum (ER). Failure to replace old or impaired organelles affects their morphology and functions. Morphological features associated with aging or senescence include the expansion of lysosomal compartments, increase of autophagic vesicles, and the presence of enlarged lysosomes containing lipofuscin, composed of highly oxidized cross-linked macromolecules that are resistant to proteolytic activity of lysosomes and clearance by exocytosis [145]. Accumulation of dysfunctional or damaged biomolecules and organelles may further interfere with proteostasis network, especially in long-lived post-mitotic cells, such as neurons, where damaged molecules and machinery cannot be diluted through mitotic cycles [15,145,146]. 

### 4.2. LRRK2 in Autophagy-Lysosome Systems

Macroautophagy, chaperone-mediated autophagy (CMA), and microautophagy are the three major forms of autophagy identified so far [147]. Macroautophagy starts with the formation of a cup-shaped membrane, phagophore that elongates and sequesters a portion to the cytoplasm to form the autophagosome. The autophagosome then fuses with the lysosome to form the autophagolysosome where the contents are degraded. In CMA, proteins are targeted to lysosomes by a chaperone through the interaction between the chaperone and a pentapeptide present in the substrate. Substrate proteins then bind to a transmembrane receptor, lysosome-associated membrane protein type 2A (LAMP-2A), which multimerizes to form the translocation complex that carries the substrate proteins into the lysosome for degradation. In microautophagy, cytoplasmic contents are directly engulfed into lysosomes for degradation. Central to all three forms of autophagy is the lysosome. Lysosome function decreases with age, with a rise in lysosomal pH [148]. A role of lysosomes in PD pathology is reflected in the PD-associated genes involved in lysosomal function, such as *VPS35*, *ATP13A2* and *GBA* [6,149]. 

Several pathogenic LRRK2 mutant models, including neuronal cell lines, primary cortical neurons and astrocytes, and Tg animal models, have reported abnormal lysosomal phenotypes, such as abnormal morphology [16,150,151,152,153], altered pH [154,155], and diminished activity [16,154]. Primary cortical neurons overexpressing LRRK2 G2019S or LRRK2 I2020T contained multivesicular bodies (MVBs) and swollen lysosomes [156]. Primary cortical neurons from bacterial artificial chromosome (BAC) Tg rats expressing LRRK2 R1441C showed decreased lysosomal acidity and alterations in lysosomal calcium dynamics [155]. Lysosomal activity depends on lysosomal pH, and acidic lysosomal pH and local calcium release from lysosomes are critical for late endosome- and autophagosome-lysosome fusion [157,158]. Interaction between LRRK2 and the a1 subunit of the v-type H+ ATPase proton pump (vATPase a1) was reduced in LRRK2 R1441C compared to WT LRRK2, leading to the dysregulation of lysosomal pH [155]. Primary cortical neurons derived from LRRK2 G2019S KI mice had abnormal lysosomal phenotypes, such as increased number of lysosomes, reduction in size, decreased lysosomal acidification, and low expression of LAMP1 [154]. Such lysosomal dysfunctions were associated with the accumulation of insoluble α-synuclein and increased release of α-synuclein, which were reversed by LRRK2 kinase inhibitors. In in vitro assays, lysates of cells expressing LRRK2 G2019S, but not LRRK2 WT, inhibited the activities of cathepsin B and L, essential lysosomal enzymes involved in the degradation of α-synuclein [159,160]. In vivo studies reported enlarged vacuolar structures with multiple membranes resembling autophagic vacuoles (AVs) as well as an accumulation of AVs in LRRK2 G2019S Tg and, to a lesser degree, in aged R1441C Tg mice [130]. A decrease in autophagic flux, an increase in p62 levels, and an accumulation of autophagosomes and lipid droplets have also been described in long-term cultures of iPSC-derived DA neurons from idiopathic PD patients and LRRK2 G2019S patients, but not in iPSC-derived DA neurons from healthy individuals [161]. 

**Figure 4:**
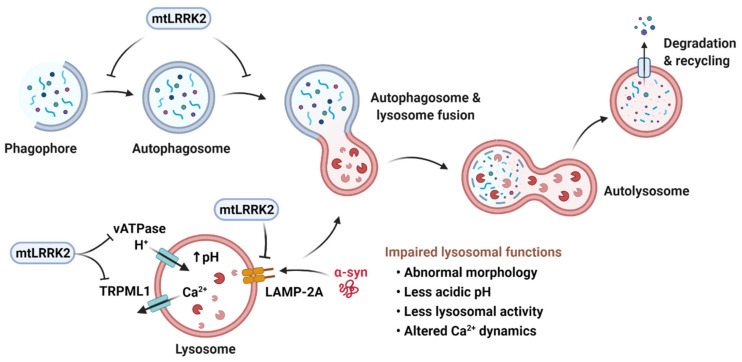
Pathogenic LRRK2 mutants (mtLRRK2) and the autophagy-lysosomal pathways. Macroautophagy is initiated by the formation of a cup-shaped membrane, termed phagophore, which engulfs damaged organelles or misfolded proteins to form the autophagosome. The autophagosome fuses with the lysosome and generates the autolysosome, in which the contents are degraded. Transition into autophagosome and autolysosome is inhibited by mtLRRK2. In chaperone-mediated autophagy, proteins are targeted to lysosomes by a chaperone through interaction between the chaperone and a pentapeptide present within the substrate. Substrate proteins then bind to a transmembrane receptor, lysosome-associated membrane protein type 2A (LAMP-2A), which multimerizes to form the translocation complex that carries the substrate proteins into the lysosome for degradation. LAMP-2A is inhibited by mtLRRK2, leading to the accumulation of CMA substrates, such as α-synuclein (α-syn). mtLRRK2 can also decrease lysosomal acidity and disrupt lysosomal calcium dynamics by inhibiting lysosomal H+-ATPase pump, vATPase and TRPML1. Created with BioRender.com.

LRRK2 and its pathogenic mutants have been suggested to be involved in several stages of the autophagy pathways (Figure 4). Studies so far have yielded a complex picture, reporting rather inconsistent role of LRRK2 in mediating autophagic changes. In early stages of autophagy, LRRK2 has been shown to modulate the phosphorylation status of p62, which recognizes ubiquitinated cargo proteins and docks them onto the forming phagophore by binding to LC3-II. LRRK2 could phosphorylate p62 at Thr138 in the ubiquitin-binding domain and co-expression of LRRK2 G2019S and p62 increased neuronal toxicity compared to non-phosphorylatable p62 [66]. In another study [162], LRRK2 WT and LRRK2 G2019S indirectly reduced p62 phosphorylation at Ser351 and Ser403 residues, associated with the initiation of autophagy [163]. LRRK2 could also phosphorylate leucyl-tRNA synthase (LRS), which is responsible for attaching leucine to tRNALeu and activating mTORC1 [164]. Phosphorylation of LRS impaired autophagy by increasing protein misfolding and ER stress. The phagophore sequestrating cargos grow into a lipidic bilayer membrane vacuole, designated as the autophagosome. Multiple studies have shown that expression of pathogenic LRRK2 mutants increased LC3 puncta, the ratio of LC3-II/LC3-I, which is often considered as an autophagic marker, and the number and size of AVs in various cellular and animal models [131,137,155,161,164,165,166,167]. 

However, it is not clear if endogenous LRRK2 facilitates or inhibits autophagy. Neurons derived from LRRK2 KO mice showed increased LC3-II levels and autophagic influx, implicating that endogenous LRRK2 has an inhibitory role in autophagy [155,168]. In addition, LRRK2 KO rodent models show increased number and size of secondary lysosome and autolysosome-like structures, accompanied with accumulation of lipofuscin granules in kidney [169,170,171]. Such abnormal accumulation of undigested materials imply impairment in ALS activity. By contrast, other studies have suggested that overexpression of LRRK2 WT [137,172,173] or LRRK2 mutants such as LRRK2 G2019S or R1441C mutants are associated with increased autophagy [174,175]. 

Less is known about effects of LRRK2 on CMA. LRRK2 WT bears pentapeptide motifs that can be targeted by hsc70 and thus could be degraded via CMA. However, high levels of WT or LRRK2 G2019S inhibited CMA by blocking the formation of the CMA translocation complex at the lysosomal membrane. The LRRK2-mediated blockage of LAMP-2A multimerization led to the accumulation of other CMA substrates, including α-synuclein [173]. These studies suggest that impaired ALS may play a part in LRRK2 toxicity and PD pathology. 

## 5. Conclusions

Advanced age is by far the strongest risk factor for PD. Even carriers of highly penetrant, disease-causing genetic mutations require aging to manifest clinical symptoms of PD. Here we have discussed how age-dependent deteriorations in cellular function may predispose individuals to the development of PD, particularly focusing on the interplay between LRRK2 and the cellular mechanisms that control oxidative stress, mitochondrial function and dynamics, and proteostasis. Recent evidence from clinical, pathological, and biochemical studies supports the notion that major differences between PD patients and healthy elderly individuals are quantitative rather than qualitative. However, we cannot entirely exclude the possibility that PD is an active pathological process, more than just a manifestation of accelerated aging. Aging affects fundamental cellular machinery common to most, if not all, cell types, and PD is now considered as a multisystem disease that affects the central, peripheral, and autonomic nervous system, causing heterogeneous clinical symptoms. However, a fact that one cannot overlook is that PD is characterized and defined by the selective degeneration of DA neurons in the SN. We have discussed possible mechanisms that confer the vulnerability of SN DA neurons, but other PD-specific genetic and environmental risk factors might contribute to the distinct clinical and pathological features of PD.

## Data Availability

Not applicable.
